# Effect of Chitosan Properties on Immunoreactivity

**DOI:** 10.3390/md14050091

**Published:** 2016-05-11

**Authors:** Sruthi Ravindranathan, Bhanu prasanth Koppolu, Sean G. Smith, David A. Zaharoff

**Affiliations:** Department of Biomedical Engineering, University of Arkansas, Fayetteville, AR 72701, USA; ravindra@uark.edu (S.R.); bkoppolu@uark.edu (B.K.); sgs004@uark.edu (S.G.S.)

**Keywords:** chitosan, immunoreactivity, endotoxin, immune response

## Abstract

Chitosan is a widely investigated biopolymer in drug and gene delivery, tissue engineering and vaccine development. However, the immune response to chitosan is not clearly understood due to contradicting results in literature regarding its immunoreactivity. Thus, in this study, we analyzed effects of various biochemical properties, namely degree of deacetylation (DDA), viscosity/polymer length and endotoxin levels, on immune responses by antigen presenting cells (APCs). Chitosan solutions from various sources were treated with mouse and human APCs (macrophages and/or dendritic cells) and the amount of tumor necrosis factor-α (TNF-α) released by the cells was used as an indicator of immunoreactivity. Our results indicate that only endotoxin content and not DDA or viscosity influenced chitosan-induced immune responses. Our data also indicate that low endotoxin chitosan (<0.01 EU/mg) ranging from 20 to 600 cP and 80% to 97% DDA is essentially inert. This study emphasizes the need for more complete characterization and purification of chitosan in preclinical studies in order for this valuable biomaterial to achieve widespread clinical application.

## 1. Introduction

Chitosan, or β-(1-4)-linked d-glucosamine and *N*-acetyl-d-glucosamine, is widely explored for use in numerous biomedical applications. A recent PubMed search indicated that, in 2015, more than 2000 biomedical-related publications cited chitosan as a keyword. To put this in perspective, there are more studies investigating chitosan than the ubiquitous biomaterials poly (lactic-co-glycolic acid) (PLGA), poly(l-lactic acid) (PLA) and polycaprolactone (PCL) combined.

Chitosan’s popularity is a result of its versatility, availability and biocompatibility. Chitosan is frequently used in the development of novel drug delivery systems and controlled release platforms [1,2,3,4,5,6,7]. It is soluble in mildly acidic, aqueous solutions and thus, is easily formulated with variety of biopharmaceuticals from small, organic cytotoxic drugs to large, labile proteins [8,9,10,11]. In addition, due to its polycationic charge in solutions, chitosan interacts efficiently with polyanionic nucleic acids to form effective non-viral gene delivery complexes [12,13,14]. Chitosan’s polycationic charge also allows it to function in wound healing and anti-microbial applications as well [15,16,17,18,19,20]. Additionally, this positive charge enables chitosan to loosen epithelial gap junctions, making it a promising candidate in mucosal delivery applications [21,22,23,24,25,26]. Chitosan and chitosan derivatives in the form of hydrogels, sponges and films are also under investigation for use as tissue engineering scaffolds [27,28,29,30,31].

Manipulation of chitosan’s biophysical properties by changing its chemical composition further contributes to its versatility. For instance, both the molecular weight and degree of deacetylation (DDA) of chitosan can be modified via simple chemical treatments [32,33]. Controlling these parameters directly affects solubility, viscosity, charge and bioadhesion. In addition, chitosan’s accessible amine and hydroxyl functional groups allow for facile conjugation of a variety of side chain moieties for limitless customization [34].

Despite the wealth of literature describing a plethora of potential biomedical uses, data are scarce and often conflicting, with regard to the nature and strength of immune responses induced by chitosan following parenteral injection or implantation. For example, in a study evaluating the biocompatibility of chitosan in mice, chitosan scaffolds were found to induce a typical acute inflammatory response marked by mild neutrophilic infiltration which dissipated over time [35]. By 12 weeks after implantation, the number of neutrophils at the implantation site was significantly reduced [35]. Similarly, in another study, chitosan hydrogel injected subcutaneously and intraperitoneally in rats induced a lower inflammatory response than the response against Vicryl^®^ (Ethicon), a PLGA absorbable surgical suture [36]. Tissue surrounding the chitosan hydrogel was found to result in a typical wound healing response without the development of hemorrhage or necrosis. In contrast, a separate study found that chitosan could activate macrophages to secrete nitric oxide, leading to long term damage of surrounding tissues [37]. Transmission electron microscopy images of chitosan and the surrounding tissue 14 days after subcutaneous implantation in male Wistar rats, showed increased fibroblast proliferation and accumulation of collagen fibrils potentially indicating chronic inflammation and fibrosis.

Given that APCs, such as dendritic cells (DCs) and macrophages, are key regulators of immunity, understanding the effects of chitosan on these cells may shed some light on chitosan’s immunoreactivity. Unfortunately, there is no consensus regarding chitosan’s impact on APC function. For instance, it was determined that chitosan activates macrophages to produce monokines, such as colony stimulating factor and IL-1 [38,39]. In another study, chitosan-based microspheres increased the cytolytic activity of peritoneal macrophages following intraperitoneal injection [40]. Similarly, other studies showed that chitosan oligosaccharides induced the robust production of pro-inflammatory cytokines, such as TNF-α and IL-1β, by macrophages [41,42]. A contrasting study found no significant increase in the production of these cytokines when DCs were treated with chitosan [43].

Even if one assumes that chitosan is capable of activating macrophages and DCs, the mechanism of activation is not well understood. In a particular study, it was determined that oligochitosan with a polymerization degree (PD) of 7–16 upregulated the expression of major histocompatibility complex class II (MHCII) and CD86 on murine splenic CD11c^+^ DCs, increased the production of tumor necrosis factor-α (TNF-α) and resulted in the proliferation of CD4^+^ T cells. Toll-like receptor 4 (TLR4) on splenic DCs were found to play an important role since silencing the receptor reduced the expression of MHCII, CD86 and TNF-α. Additionally, these effects were observed only upon treatment with oligochitosan of PD 7–16 and not with PD of 3–7 [44]. In contrast, another study found that oligochitosan of PD 3–10 increased the production of TNF-α by mouse macrophages. Unlike the TLR4 study, mannose receptors were found to play a key role in chitosan-induced activation [45]. Another type of receptor, NOD-like receptor family pyrin domain containing 3 (NLRP3), was found to be activated by chitosan and induce the release of IL-1β [46].

Thus, it is evident from the literature that there are a number of discrepancies related to chitosan’s immunoreactivity. Chitosan is viewed by some as an inert biomaterial that induces no more than a mild, transient foreign body reaction. To others, chitosan induces a specific, inflammatory response initiated by direct molecular recognition. It is possible that both sides are correct. Chitosan is a diverse class of molecules manufactured from a variety of raw materials. Different chitosans may induce different immune responses. Moreover, some researchers also suspect most, if not all, chitosans to be contaminated with varying levels of endotoxin which would impact immune responses significantly [47]. Thus, there is a compelling need for a systematic study to resolve large, contradictory discrepancies and to gain a better understanding of the immunoreactivity of chitosan. In particular, the issue of endotoxin contamination must be addressed for chitosan to achieve widespread clinical application. For now, chitosan’s clinical use is limited to topical applications, e.g., hemostatic bandages, despite its considerable potential in other arenas.

The goal of this study was to assess the effect of chitosan’s modifiable parameters, *i.e.*, molecular weight and DDA, as well as endotoxin contamination on the function of critical APC populations *in vitro*. Findings from this study should be of interest to any investigator developing chitosan-based implants and injectables. This study also makes the case for more detailed testing and reporting of biochemical parameters when using chitosan in biomedical applications.

## 2. Results

### 2.1. Chitosan Induced Cytokine Production

**Figure 1:**
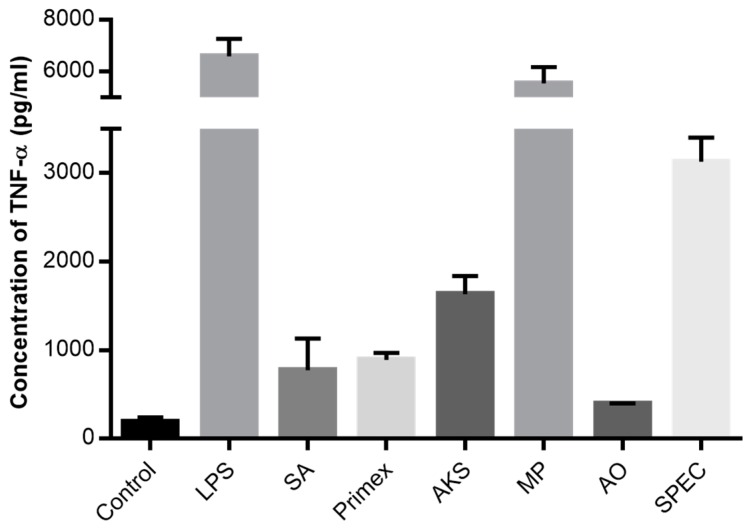
Immune response to commercially available chitosan. TNF-α released by Raw 264.7 macrophages upon 24-h incubation with 0.1 µg/mL LPS, 0.1 mg/mL of chitosan solutions from six different manufacturers; Sigma-Aldrich (SA), Primex, AK Scientific (AKS), MP Biomedicals (MP), Acros Organics (AO) and Spectrum (SPEC) or the cell medium alone as negative control. Data presented are mean with SEM from three independent measurements.

Mouse macrophages treated with chitosan from different sources secreted an enormous range of TNF-α (Figure 1). Among the six chitosan sources, the concentration of TNF-α was highest in supernatant collected from cells treated with chitosan from MP Biomedicals (5553.1 ± 373.7 pg/mL) and lowest with chitosan from Acros Organics (397.0 ± 27.1 pg/mL). Chitosan from MP Biomedicals induced a similar response as pure LPS (6606.3 ± 416.6 pg/mL). Untreated cells produced less than 200 pg/mL TNF-α. All chitosan treatments induced TNF-α levels that were significantly different from one another and higher than the untreated control group (*p* < 0.05 via ANOVA and Dunnett’s post test).

### 2.2. Characterization of Commercially Available Chitosan

Viscosity, DDA and endotoxin content in each of the six chitosan samples were measured and tabulated. As seen in Table 1, each chitosan was found to have different levels of endotoxin contamination and also greatly varied with respect to viscosity and DDA. Chitosan from Spectrum chemicals was found to be highly contaminated with endotoxin levels averaging 3.45 ± 0.04 EU/mg, whereas chitosan from Primex contained only 0.22 ± 0.06 EU/mg. The DDA ranged from 74% to 98% while viscosities of 1% chitosan solutions varied from 13 to 265 cP among the six different chitosan samples respectively.

### 2.3. Effect of DDA on Cytokine Release

**Figure 2:**
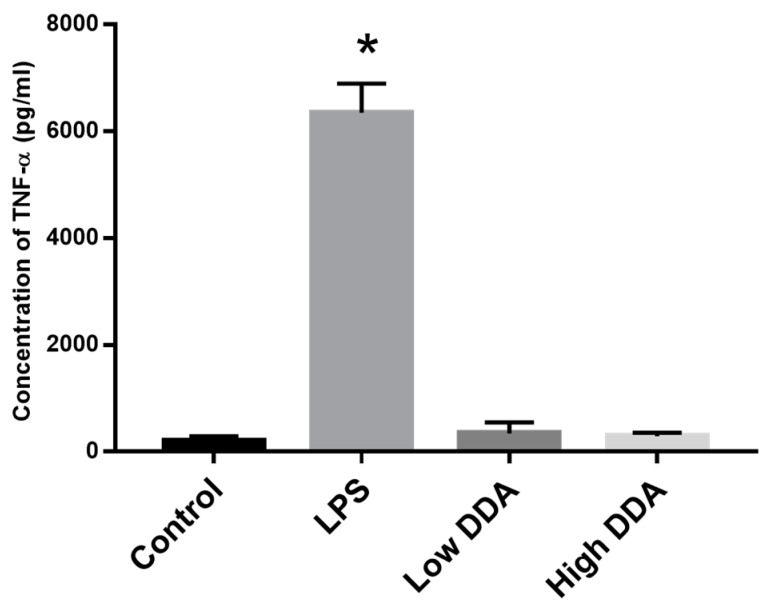
Effect of DDA on mouse macrophages. TNF-α released by mouse macrophages upon 24-h incubation with low endotoxin (<0.01 EU/mg) chitosan of 80% (Low) and 97% (High) DDA from UABC. LPS (0.1 µg/mL) and media alone served as positive and negative controls, respectively. Data presented are mean plus SEM from three independent measurements. Amount of TNF-α released upon treatment with LPS was significantly different with *p* < 0.05 and is represented with (*).

Based on the data shown in Table 1, it was not possible to correlate the effects of any of the three properties measured with TNF-α production (Figure 1). Therefore, in order to isolate the effect of DDA on cytokine release, custom purified chitosan of two different DDAs, 80% and 97%, but of same viscosity (95 ± 25 cP) were obtained from UABC. This purified chitosan contained undetectable (<0.01 EU/mg) levels of endotoxin. Upon incubation with mouse macrophages, no significant difference was found in the TNF-α released between the two chitosan samples (Figure 2). The amount of TNF-α released in both cases was similar to media alone control group, 193.7 ± 67.5 pg/mL (*p* > 0.05 via ANOVA and Dunnett’s post test).

### 2.4. Effect of Viscosity on Cytokine Release

**Figure 3:**
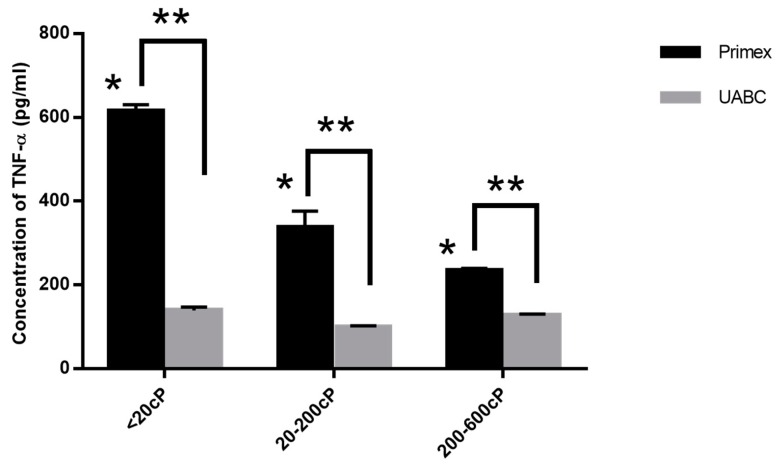
Effect of endotoxin and viscosity on immune response of chitosan. TNF-α released by RAW 264.7 macrophages upon 24 h incubation with chitosans with viscosities of <20 cP, 20–200 cP and 200–600 cP from Primex and UABC. Data presented are mean plus SEM from three independent measurements. * *p* < 0.05 for Primex chitosan of viscosities <20 cP, 20–200 cP and 200–600 cP. ** *p* < 0.05 for Primex *vs.* UABC.

To isolate the effect of viscosity or polymer chain length, RAW 264.7 macrophages were treated with chitosans from both Primex and UABC with three different viscosity ranges, <20 cP, 20–200 cP and 200–600 cP, at a single DDA of 80%. Significant differences in TNF-α production between the different viscosity samples from the Primex chitosan was observed (Figure 3). However, when purified chitosan from UABC was used, no significant difference was found in the TNF-α release between samples of different viscosities (*p* > 0.05 via ANOVA). Additionally, for UABC chitosans, the amount of TNF-α release was indistinguishable from the untreated control group, 128 ± 17.5 pg/mL (*p* > 0.05 via ANOVA and Dunnett’s post test).

### 2.5. Effect of Endotoxin Contamination on Cytokine Release

**Figure 4:**
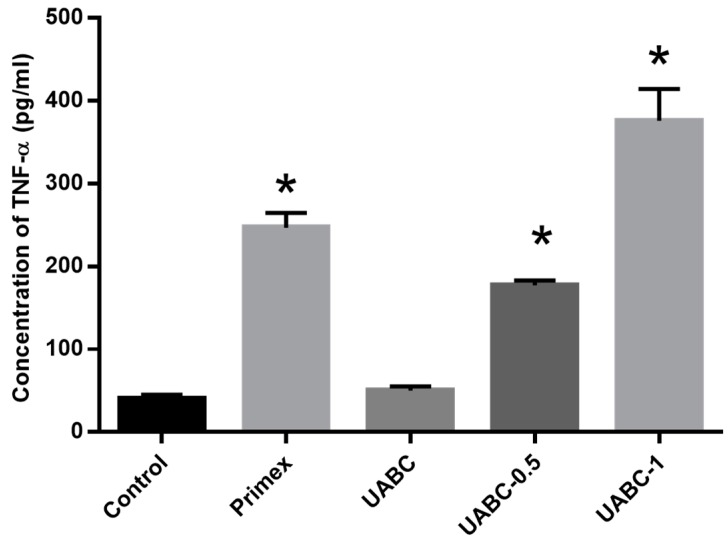
Effect of endotoxin content on immune response of chitosan. TNF-α released by mouse macrophages upon 24-h incubation with Primex, UABC and UABC chitosan spiked with 0.5 or 1 EU (UABC−0.5/UABC 1). Cells treated with media alone served as negative control. Data presented are mean plus SEM from three independent measurements. * *p* < 0.05 *vs.* control via ANOVA and Tukey’s *post hoc* test.

To determine if endotoxin content is directly responsible for immunoreactivity, UABC chitosan spiked in with varying levels of endotoxin (0.5 and 1 EU) were exposed to RAW 264.7 cells. Both spiked samples elicited significantly higher levels of TNF-α when compared to the untreated control (Figure 4). In fact, chitosan spiked with 1 EU released higher levels of TNF-α (376 ± 38 pg/mL) when compared to chitosan with 0.5 EU (177 ± 5 pg/mL). Here, the amount of TNF released by the cells upon treatment with media alone or LPS were 39 ± 5 pg/mL and 12,700 ± 251 pg/mL respectively.

### 2.6. Effect of Endotoxin Contamination on Mouse Dendritic Cells

**Figure 5:**
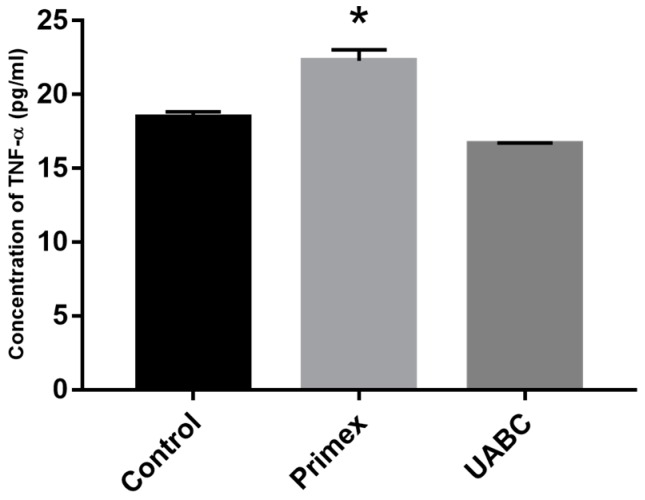
Effect of endotoxin on BMDCs. TNF-α released by BMDCs upon 24 h incubation with chitosan from Primex and UABC. Data presented are mean plus SEM from three independent measurements. Media alone served as negative control. * *p* < 0.05 *vs.* control and UABC via Tukey’s *post hoc* analysis.

To verify that differences in chitosan-induced immunoreactivity were not an artifact of RAW 264.7 macrophages, chitosans from both Primex and UABC were exposed to BMDCs isolated from healthy mouse femurs. Chitosans from Primex and UABC were similar in viscosity, 20–200 cP, and DDA, 80%, however, endotoxin levels were 0.22 ± 0.06 EU/mg and <0.01 EU/mg, respectively. Chitosan from Primex induced significantly higher levels of TNF-α compared to purified chitosan from UABC −29.0 ± 6.7 pg/mL *vs.* 14.9 ± 1.8 pg/mL (Figure 5). Once again the response to UABC chitosan was similar to untreated controls (*p* > 0.05 via Tukey’s post test). LPS-induced TNF-α secretion by BMDCs was orders of magnitude higher at 2597.2 ± 76.2 pg/mL (data not shown on graph).

### 2.7. Effect of Endotoxin Contamination on Human Macrophage Cell Line

**Figure 6:**
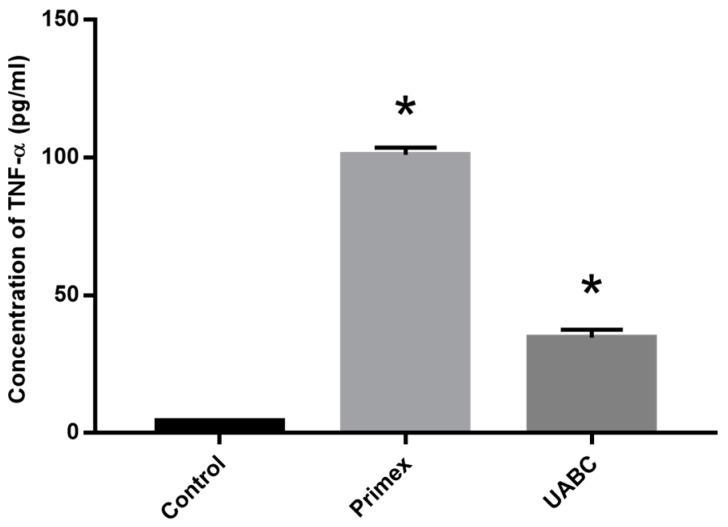
Effect of endotoxin contamination of chitosan on human macrophage cell line. Human macrophages were differentiated from THP1 cells and treated with chitosan from Primex and UABC or media alone (control). Data presented are mean plus SEM from three independent measurements. * *p* < 0.05 *vs.* control.

To confirm that differences in chitosan-induced immunoreactivity were not restricted to murine cells, human macrophages differentiated from THP1 cells were treated with chitosan (80% DDA and 20–200 cP viscosity) from Primex and UABC as above (Figure 6). The amount of TNF-α produced by THP1 cells exposed to Primex chitosan, 101.1 ± 2.6 pg/mL, was significantly higher than TNF-α following exposure to the UABC chitosan, 34.7 ± 2.8 pg/mL (*p* < 0.05 via Tukey’s post test). With media alone and LPS, THP1 cells produced 4.3 ± 0.4 pg/mL and 4522.7 ± 149.9 pg/mL TNF-α, respectively.

## 3. Discussion

Chitosan is a promising, versatile biopolymer with numerous potential clinical applications. However, there is no clear consensus regarding the immunoreactivity of chitosan. This is partly due to a lack of standardization in chitosan-based studies. In a recent review, it was correctly noted that most studies involving chitosan fail to fully disclose important characteristics such as the molecular weight, DDA, source and purity of chitosan [48]. As a result, it is difficult to compare results between groups which use chitosan from different sources. Our characterization studies showed that there were indeed large differences in chitosans from different manufacturers in terms of viscosity, DDA and endotoxin contamination (Table 1).

Because chitosan is a natural biopolymer produced primarily via multiple cycles of alkaline and acidic treatments of crustacean exoskeletons, some variation in physico-chemical properties can be expected. Different treatment conditions inevitably lead to differences in molecular weights/viscosities and DDAs. As a result, chitosan from a single source can vary from batch-to-batch.

To understand the effects of chitosan’s properties on immunoreactivity, our studies focused on cellular immune responses to chitosan. By focusing on the cell level, we are able to isolate chitosan-specific immunoreactivity from more complex processes, such as foreign body reactions and inflammatory responses that occur following *in vivo* administrations. In particular, professional APCs such as macrophages and dendritic cells are key mediators of immunity. Thus, understanding how these APCs respond to different chitosans is essential for helping resolve discrepancies in the literature and supporting clinical translation of chitosan-based applications.

TNF-α was used as an indicator of immunoreactivity since it is an important early signaling protein and a highly sensitive indicator of immune responsiveness. Our preliminary studies which also measured IL-1β and IL-6 levels in response to chitosan exposure, demonstrated that these cytokines were not as sensitive as TNF-α (data not shown).

After the initial characterization (Table 1) and TNF-α release (Figure 1) studies, it was clear that (1) immune responses were highly variable; and (2) it was not possible to isolate the specific effects of viscosity, DDA and endotoxin contamination using commercially available chitosans. In addition, despite endotoxins having a major effect on chitosan immunoreactivity, it was not possible to correlate endotoxin levels in the different chitosans with TNF-α release. This is due to the fact that the activity of endotoxins vary widely with the intrinsic activity of its lipid associated protein [49]. Because these chitosans were derived from different sources, the types of endotoxin were likely to be different. Nevertheless, in anticipation that endotoxin contamination would be a significant factor in driving the immune response, we obtained, from the UABC, purified chitosan that contained undetectable levels of endotoxin. This purified chitosan allowed us to uncouple the effects of molecular weight, DDA and endotoxin contamination.

By treating mouse macrophages with chitosans of different viscosities and DDAs but with the same amount of endotoxin (<0.01 EU/mg), we found that neither DDA (Figure 2) nor viscosity/molecular weight (Figure 3) had any effect on the immune response. Additionally, by spiking in known amounts of endotoxin into UABC chitosan, we confirmed that the immunoreactivity is directly affected by amount of endotoxin contamination in chitosan.

Regarding the effect of viscosity/molecular weight, it was previously shown that higher molecular weight oligochitosans, PD 7–16, were more immunoreactive, in terms of upregulation of antigen presenting and co-stimulatory molecules as well as TNF-α production, than lower molecular weight oligochitosans, PD 3–7 [26]. We estimate that even our smallest chitosan had a PD of about 20–40 which is much higher than the oligochitosans of previous studies. As a result, it is possible that viscosity/molecular weight does not impact immunoreactivity above a threshold chain length. It is also possible that oligochitosans used in previous studies contained varying amounts of endotoxin which, as we have shown above, can lead to significant differences in immunoreactivity. Endotoxin levels were not reported in the oligochitosan study [26]. Nevertheless, planned studies will explore smaller, purified oligochitosans to determine if we find the same effect of molecular weight with smaller chitosans.

Regarding the effect of DDA, it was previously shown that chitosan scaffolds of 85% DDA led to greater mononuclear cell infiltration as compared to 96% DDA [50]. Furthermore, the density of infiltrates increased over a 2-week period for the lower DDA scaffold. While our study found no effect of DDA, we looked at a simple system aimed at understanding cellular responses and not *in vivo* foreign body response. It is possible that immune responses may change *in vivo* when more cells and processes are involved. This is the subject of future studies.

It is important to reiterate that chitosan-induced responses were not restricted to a single cell line or species. Similar results were obtained with RAW 264.7 murine macrophages and murine BMDCs. Since mice are generally less sensitive to endotoxins than humans, it was also useful to observe that human macrophages differentiated from THP1 similarly responded to endotoxin contamination in chitosan by producing higher levels of TNF-α (Figure 4). While it is not surprising that higher endotoxin levels lead to higher pro-inflammatory cytokine production, these data emphasize the need for chitosan-based preclinical studies to provide a complete biochemical characterization, including endotoxin levels, of any chitosans used.

## 4. Materials and Methods

### 4.1. Reagents

Chitosan was obtained from Sigma-Aldrich (St. Louis, MO, USA), Acros Organics (Bridgewater, NJ, USA), AK Scientific (Union City, CA, USA), MP Biomedicals (Solon, Ohio, OH, USA), Spectrum Chemical Mfg Corp. (New Brunswick, NJ, USA) and Primex (Siglufjordor, Iceland). Purified, low endotoxin chitosan (<0.01 EU/mg) was obtained from the University of Arkansas Biologics Center (UABC) (Fayetteville, AR, USA). Sodium hydroxide was purchased from Thermo Fisher Scientific Inc. (Waltham, MA, USA). Hydrochloric acid and acetic acid were purchased from Sigma-Aldrich (St. Louis, MO, USA). Unless otherwise specified, chitosan solutions were prepared by dissolving chitosan in 0.1 M HCl and adjusted to pH 6.0 using NaOH.

Cell culture media components including Dulbecco’s modified Eagle’s medium (DMEM), Roswell Park Memorial Institute 1640 (RPMI-1640), fetal bovine serum (FBS), l-glutamine and penicillin-streptomycin solution were purchased from Hyclone Laboratories (Logan, UT, USA). Ammonium-chloride-potassium (ACK) buffer used in the process of isolating bone marrow derived dendritic cells from mouse was purchased from Lonza (Allendale, NJ, USA). Recombinant murine granulocyte-macrophage colony stimulating factor (rmGM-CSF) was purchased from Peprotech (Rocky Hill, NJ, USA). Lipopolysaccharide (LPS) from Salmonella enterica serotype enteritidis was purchased from Sigma-Aldrich.

### 4.2. Laboratory Animals

Female C57BL/6 J mice were purchased from The Jackson Laboratory (Bar Harbor, ME, USA). Mice were housed in microisolator cages and used at 8–12 weeks of age. All experimental procedures were approved by the Institutional Animal Care and Use Committee at the University of Arkansas. Animal care was in compliance with The Guide for Care and Use of Laboratory Animals (National Research Council).

### 4.3. Cell Culture

RAW 264.7 murine macrophages and THP1 human monocyte leukemia cells were purchased from American Type Culture Collection (Manassas, VA, USA). RAW 264.7 macrophages were cultured in Dulbecco’s Modified Eagle Medium (DMEM) with 10% fetal bovine serum (FBS) and 1% penicillin/streptomycin. THP1 cells were differentiated into macrophages and cultured in RPMI-1640 with 10% FBS as described previously [51]. Bone marrow derived dendritic cells were isolated from C57BL/6 mice, as described elsewhere [52]. All cultures were maintained in a humidified CO_2_ incubator at 37 °C and 5% CO_2_.

### 4.4. Determination of Relative Viscosity, DDA and Endotoxin Content

Relative viscosity was used as a surrogate for polymer chain length or molecular weight. A 1% (*w*/*v*) solution of each chitosan was prepared in 1% (*v*/*v*) acetic acid (Sigma-Aldrich) and the viscosity was measured using an LV DVIII rheometer from Brookfield Engineering Laboratories (Middleboro, MA, USA). Readings were taken at 25 °C using a CP-40 spindle. The DDA of the chitosan solutions were determined, as described previously [32]. Recombinant Factor C assay (rFC) assay from Lonza was used to measure the level of endotoxin in chitosan solutions following the procedure described by the manufacturer.

### 4.5. Measurement of Immune Responses

RAW 264.7 macrophages were exposed to 0.1 mg/mL of each chitosan solution for 24 h. The amount of TNF-α in cell culture supernatants was quantified via enzyme linked immunosorbent assay (ELISA) kits from eBiosciences (San Diego, CA, USA) following the protocol provided by the manufacturer. Cells treated with 0.1 µg/mL LPS or media alone served as positive and negative controls, respectively. Endotoxin standard (*E.coli*, O55:B5) provided in the rFC assay kit was spiked into UABC chitosan to confirm its effect on immunoreactivity.

### 4.6. Statistical Analysis

All experiments were performed in triplicate. Data are reported as mean ± standard error of the mean (SEM) or standard deviation. Student’s *t*-test was used to compare two groups of interest. For more than two groups, analysis of variance (ANOVA) was performed followed by Tukey’s or Dunnett’s post test analyses. Statistical differences were accepted at the *p* < 0.05 level.

## 5. Conclusions

Despite its remarkable potential for use in a range of medical applications, discrepancies in chitosan’s immunoreactivity have limited its clinical use. By exploring the direct effects of chitosan on immune responses at the cellular level, we are able to isolate chitosan-specific immunoreactivity from more complex processes such as foreign body reactions and inflammatory responses that occur following injections *in vivo*. Initial characterization studies demonstrated that commercially available chitosans, in solution, induce varying degrees of immune responses. Using purified, low endotoxin chitosan, it was determined that viscosity/molecular weight and DDA within the ranges 20–600 CP and 80%–97%, respectively, have no impact on chitosan’s immunoreactivity. Similar results were found using murine and human macrophages as well as murine dendritic cells. In retrospect, large differences in immune responses, both in our initial characterization of commercially available chitosan as well as results found in the literature, may be explained by highly variable endotoxin levels. It should be noted that because immunoreactivity was assessed solely based on TNF-α production, to fully appreciate the immunoreactivity of chitosan, *in vitro* studies characterizing other inflammatory cytokines, such as IL-1 and IL-6, as well as *in vivo* studies using purified chitosans must be performed. Ultimately, endotoxin contamination is expected to remain as the chief factor influencing immunoreactivity. Therefore, we suggest that endotoxin levels be explicitly reported in all future biomedical studies involving chitosan. It should also be noted that chitosan solutions were evaluated in this study. While we expect to find similar low reactivity to chitosan in solid forms, such as particles, scaffolds, films, *etc.*, no conclusions can be drawn without additional experimentation.

## Figures and Tables

**Table 1:** Comparison of properties of commercially available chitosan. The amount of endotoxin contamination, degree of deacetylation (DDA) and viscosity for each of the six different commercially available chitosan considered in this study were measured as described. Data presented are mean ± standard deviation from three independent measurements.

Chitosan	Molecular Weight Provided by Manufacturer (kDa)	Amount of Endotoxin (EU/mg)	DDA (%)	Viscosity (cP)
Sigma-Aldrich	50–190	1.48 ± 0.05	74 ± 0.1	265 ± 5
Primex	Not provided	0.22 ± 0.06	80 ± 0.3	95 ± 25
AK Scientific	Not provided	1.44 ± 0.03	96 ± 0.4	65 ± 20
MP Biomedicals	Not provided	2.27 ± 0.03	88 ± 0.9	19 ± 6
Acros Organics	100–300	1.09 ± 0.05	98 ± 0.4	58 ± 16
Spectrum chemicals	Not provided	3.45 ± 0.04	92 ± 0.4	13 ± 5

## References

[1] Agnihotri S.A., Mallikarjuna N.N., Aminabhavi T.M. (2004). Recent advances on chitosan-based micro-and nanoparticles in drug delivery. J Control. Release.

[2] Al Rubeaan K., Rafiullah M., Jayavanth S. (2016). Oral insulin delivery systems using chitosan-based formulation: A review. Expert Opin. Drug Deliv..

[3] Dang Q., Liu C., Wang Y., Yan J., Wan H., Fan B. (2016). Characterization and biocompatibility of injectable microspheres-loaded hydrogel for methotrexate delivery. Carbohydr. Polym..

[4] Park J.H., Saravanakumar G., Kim K., Kwon I.C. (2010). Targeted delivery of low molecular drugs using chitosan and its derivatives. Adv. Drug Deliv. Rev..

[5] Risbud M.V., Hardikar A.A., Bhat S.V., Bhonde R.R. (2000). pH-sensitive freeze-dried chitosan—Polyvinyl pyrrolidone hydrogels as controlled release system for antibiotic delivery. J. Control. Release.

[6] Ruel-Gariepy E., Leclair G., Hildgen P., Gupta A., Leroux J. (2002). Thermosensitive chitosan-based hydrogel containing liposomes for the delivery of hydrophilic molecules. J. Control. Release.

[7] Vo J.L., Yang L., Kurtz S.L., Smith S.G., Koppolu B.P., Ravindranathan S., Zaharoff D.A. (2014). Neoadjuvant immunotherapy with chitosan and interleukin-12 to control breast cancer metastasis. Oncolmmunology.

[8] Gan Q., Wang T. (2007). Chitosan nanoparticle as protein delivery carrier—Systematic examination of fabrication conditions for efficient loading and release. Colloids Surf. B Biointerfaces.

[9] Koppolu B., Zaharoff D.A. (2013). The effect of antigen encapsulation in chitosan particles on uptake, activation and presentation by antigen presenting cells. Biomaterials.

[10] Mehrotra A., Nagarwal R.C., Pandit J.K. (2011). Lomustine loaded chitosan nanoparticles: Characterization and *in vitro* cytotoxicity on human lung cancer cell line L132. Chem. Pharm. Bull..

[11] Koppolu B., Smith S.G., Ravindranathan S., Jayanthi S., Kumar T.K.S., Zaharoff D.A. (2014). Controlling chitosan-based encapsulation for protein and vaccine delivery. Biomaterials.

[12] Bao H., Pan Y., Ping Y., Sahoo N.G., Wu T., Li L., Li J., Gan L.H. (2011). Chitosan-functionalized graphene oxide as a nanocarrier for drug and gene delivery. Small.

[13] Lu H., Dai Y., Lv L., Zhao H. (2014). Chitosan-graft-polyethylenimine/DNA nanoparticles as novel non-viral gene delivery vectors targeting osteoarthritis. PLoS ONE.

[14] Raftery R., O’Brien F.J., Cryan S. (2013). Chitosan for gene delivery and orthopedic tissue engineering applications. Molecules.

[15] Charernsriwilaiwat N., Opanasopit P., Rojanarata T., Ngawhirunpat T. (2012). Lysozyme-loaded, electrospun chitosan-based nanofiber mats for wound healing. Int. J. Pharm..

[16] Dai T., Tanaka M., Huang Y., Hamblin M.R. (2011). Chitosan preparations for wounds and burns: Antimicrobial and wound-healing effects. Expert Rev. Anti-Infect. Ther..

[17] Kweon D., Song S., Park Y. (2003). Preparation of water-soluble chitosan/heparin complex and its application as wound healing accelerator. Biomaterials.

[18] Lih E., Lee J.S., Park K.M., Park K.D. (2012). Rapidly curable chitosan—PEG hydrogels as tissue adhesives for hemostasis and wound healing. Acta Biomater..

[19] Moura L.I., Dias A.M., Leal E.C., Carvalho L., de Sousa H.C., Carvalho E. (2014). Chitosan-based dressings loaded with neurotensin—An efficient strategy to improve early diabetic wound healing. Acta Biomater..

[20] Wang B., Liu X., Ji Y., Ren K., Ji J. (2012). Fast and long-acting antibacterial properties of chitosan-Ag/polyvinylpyrrolidone nanocomposite films. Carbohydr. Polym..

[21] Casettari L., Illum L. (2014). Chitosan in nasal delivery systems for therapeutic drugs. J. Control. Release.

[22] Kumar M., Behera A.K., Lockey R.F., Zhang J., Bhullar G., de la Cruz C.P., Chen L.C., Leong K.W., Huang S.K., Mohapatra S.S. (2002). Intranasal gene transfer by chitosan-DNA nanospheres protects BALB/c mice against acute respiratory syncytial virus infection. Hum. Gene Ther..

[23] Luo Y., Wang Q. (2014). Recent development of chitosan-based polyelectrolyte complexes with natural polysaccharides for drug delivery. Int. J. Biol. Macromol..

[24] Smith S.G., Koppolu B., Ravindranathan S., Kurtz S.L., Yang L., Katz M.D., Zaharoff D.A. (2015). Intravesical chitosan/interleukin-12 immunotherapy induces tumor-specific systemic immunity against murine bladder cancer. Cancer Immunol. Immunother..

[25] Ven der Lubben I.M., Verhoef J.C., Borchard G., Junginger H.E. (2001). Chitosan and its derivatives in mucosal drug and vaccine delivery. Eur. J. Pharm. Sci..

[26] Yao W., Peng Y., Du M., Luo J., Zong L. (2013). Preventative vaccine-loaded mannosylated chitosan nanoparticles intended for nasal mucosal delivery enhance immune responses and potent tumor immunity. Mol. Pharm..

[27] Bhardwaj N., Kundu S.C. (2012). Chondrogenic differentiation of rat MSCs on porous scaffolds of silk fibroin/chitosan blends. Biomaterials.

[28] Jana S., Florczyk S.J., Leung M., Zhang M. (2012). High-strength pristine porous chitosan scaffolds for tissue engineering. J. Mater. Chem..

[29] Levengood S.K.L., Zhang M. (2014). Chitosan-based scaffolds for bone tissue engineering. J. Mater. Chem. B.

[30] Mukhopadhyay P., Sarkar K., Bhattacharya S., Bhattacharyya A., Mishra R., Kundu P. (2014). pH sensitive *N*-succinyl chitosan grafted polyacrylamide hydrogel for oral insulin delivery. Carbohydr. Polym..

[31] Nettles D.L., Elder S.H., Gilbert J.A. (2002). Potential use of chitosan as a cell scaffold material for cartilage tissue engineering. Tissue Eng..

[32] Yuan Y., Chesnutt B.M., Haggard W.O., Bumgardner J.D. (2011). Deacetylation of chitosan: Material characterization and *in vitro* evaluation via albumin adsorption and pre-osteoblastic cell cultures. Materials.

[33] Mao S., Shuai X., Unger F., Simon M., Bi D., Kissel T. (2004). The depolymerization of chitosan: Effects on physicochemical and biological properties. Int. J. Pharm..

[34] Riva R., Ragelle H., des Rieux A., Duhem N., Jérôme C., Préat V. (2011). Chitosan and chitosan derivatives in drug delivery and tissue engineering. Chitosan for Biomaterials II.

[35] Vande Vord P.J., Matthew H.W.T., DeSilva S.P., Mayton L., Wu B., Wooley P.H. (2002). Evaluation of the biocompatibility of a chitosan scaffold in mice. J. Biomed. Mater. Res..

[36] Azab A.K., Doviner V., Orkin B., Kleinstern J., Srebnik M., Nissan A., Rubinstein A. (2007). Biocompatibility evaluation of crosslinked chitosan hydrogels after subcutaneous and intraperitoneal implantation in the rat. J. Biomed. Mater. Res. A.

[37] Peluso G., Petillo O., Ranieri M., Santin M., Ambrosic L., Calabro D., Avallone B., Balsamo G. (1994). Chitosan mediated stimulation of macrophage function. Biomaterials.

[38] Suzuki K., Okawa Y., Hashimoto K., Suzuki S., Suzuki M. (1984). Protecting effect of chitin and chitosan on experimentally induced murine candidiasis. Microbiol. Immunol..

[39] Nishimura K., Ishihara C., Ukei S., Tokura S., Azuma I. (1986). Stimulation of cytokine production in mice using deacetylated chitin. Vaccine.

[40] Nishimura K., Nishimura S., Seo H., Nishi N., Tokura S., Azuma I. (1987). Effect of multiporous microspheres derived from chitin and partially deacetylated chitin on the activation of mouse peritoneal macrophages. Vaccine.

[41] Feng J., Zhao L., Yu Q. (2004). Receptor-mediated stimulatory effect of oligochitosan in macrophages. Biochem. Biophys. Res. Commun..

[42] Chen C., Wang Y., Liu C., Wang J. (2008). The effect of water-soluble chitosan on macrophage activation and the attenuation of mite allergen-induced airway inflammation. Biomaterials.

[43] Villiers C., Chevallet M., Diemer H., Couderc R., Freitas H., Van Dorsselaer A., Marche P.N., Rabilloud T. (2009). From secretome analysis to immunology: Chitosan induces major alterations in the activation of dendritic cells via a TLR4-dependent mechanism. Mol. Cell Proteom..

[44] Dang Y., Li S., Wang W., Wang S., Zou M., Guo Y., Fan J., Du Y., Zhang J. (2011). The effects of chitosan oligosaccharide on the activation of murine spleen CD11c dendritic cells via Toll-like receptor 4. Carbohydr. Polym..

[45] Han Y., Zhao L., Yu Z., Feng J., Yu Q. (2005). Role of mannose receptor in oligochitosan-mediated stimulation of macrophage function. Int. Immunopharmacol..

[46] Bueter C.L., Lee C.K., Rathinam V.A., Healy G.J., Taron C.H., Specht C.A., Levitz S.M. (2011). Chitosan but not chitin activates the inflammasome by a mechanism dependent upon phagocytosis. J. Biol. Chem..

[47] Lieder R., Gaware V.S., Thormodsson F., Einarsson J.M., Ng C.H., Gislason J., Masson M., Petersen P.H., Sigurjonsson O.E. (2013). Endotoxins affect bioactivity of chitosan derivatives in cultures of bone marrow-derived human mesenchymal stem cells. Acta Biomater..

[48] Vasiliev Y.M. (2014). Chitosan-based vaccine adjuvants: Incomplete characterization complicates preclinical and clinical evaluation. Expert Rev. Vaccines.

[49] Morrison D.C., Ulevitch R.J. (1978). The effects of bacterial endotoxins on host mediation systems. A review. Am. J. Pathol..

[50] Barbosa J.N., Amaral I.F., Aguas A.P., Barbosa M.A. (2010). Evaluation of the effect of the degree of acetylation on the inflammatory response to 3D porous chitosan scaffolds. J. Biomed. Mater. Res. A.

[51] Schwende H., Fitzke E., Ambs P., Dieter P. (1996). Differences in the state of differentiation of THP-1 cells induced by phorbol ester and 1,25-dihydroxyvitamin D3. J. Leukoc. Biol..

[52] Inaba K., Inaba M., Romani N., Aya H., Deguchi M., Ikehara S., Muramatsu S., Steinman R.M. (1992). Generation of large numbers of dendritic cells from mouse bone marrow cultures supplemented with granulocyte/macrophage colony-stimulating factor. J. Exp. Med..

